# Leveraging explainable machine learning models to assess forest health: A case study in Hainan, China

**DOI:** 10.1002/ece3.10558

**Published:** 2023-09-25

**Authors:** Jialing Li, Bohao He, Shahid Ahmad, Wei Mao

**Affiliations:** ^1^ School of Ecology and Environment Hainan University Haikou China; ^2^ Key Laboratory of Agro‐Forestry Environmental Processes and Ecological Regulation of Hainan Province Hainan University Haikou China; ^3^ Wuzhishan Division Hainan Tropical Rainforest National Park Bureau Wuzhishan China

**Keywords:** CRITIC method, decision tree modeling, forest health assessment, forest health indicators, Hainan tropical rainforest national park, tropical rainforest health

## Abstract

Global forest area has declined over the past few years, forest quality has declined, and ecological and environmental events have increased with climate change and human activity. In the context of ecological civilization, forest health issues have received unprecedented attention. By improving forest health, forests can better perform their ecosystem service functions and promote green development. This study was carried out in the WuZhi Shan area of Hainan Tropical Rainforest National Park. We employed a decision tree algorithm, a machine learning technique, for our modeling due to its high accuracy and interpretability. The objective weighted method using criteria of importance through intercriteria correlation (CRITIC) was used to determine forest health classes based on survey and experimental data from 132 forest samples. The results showed that species diversity is the most important metric to measure forest health. An interpretable decision tree machine learning model was proposed to incorporate forest health indicators, providing up to 90% accuracy in the classification of forest health conditions. The model demonstrated a high degree of effectiveness, achieving an average precision of 90%, a recall of 67%, and an F1 score of 70.2% in predicting forest health. The interpretable decision tree classification results showed that breast height diameter is the most important variable in classifying the health status of both primary and secondary forests. This study highlights the importance of using interpretable machine learning methods for the decision‐making process. Our work contributes to the scientific underpinnings of sustainable forest development and effective conservation planning.

## Graphical abstract

Machine learning can be used to classify whether forest health is healthy or not. This study highlights the importance of using interpretable machine learning methods for the decision‐making process. Our work contributes to the scientific underpinnings of sustainable forest development and effective conservation planning.

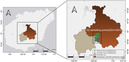

## 1 INTRODUCTION

Forest ecosystems are the most widespread terrestrial ecosystem and biologically the largest vegetation type (Pan et al., 2013), providing human beings with a treasure trove of resources that play a vital role in global energy flow, material recycling, and information transfer (De Frenne et al., 2021; Messier et al., 2019). Forest health, a comprehensive reflection of the vitality and sustainability of a forest ecosystem, is critical to maintaining the stability of the global environment (Trumbore et al., 2015). It also provides direct and indirect ecological services to humans (Gilhen‐Baker et al., 2022). It provides timber, crops, oils, and medicinal resources that improve the socioeconomic condition of local residents (Kenter et al., 2015), regulate the balance of oxygen and carbon dioxide in the biosphere, promote water conservation, air purification, and protect biodiversity (Liang et al., 2016; Watson et al., 2018). A forest ecosystem is a natural complex with a certain structure, function, and self‐regulation formed by a forest community and its environment under the influence of functional flow, and it is the largest natural ecosystems in the terrestrial landscape (Czembrowski & Kronenberg, 2016; Watson et al., 2018).

Air pollutants, pests, diseases, global climate change, and the human footprint are all contributing to varying degrees of forest degradation, and forest ecosystem health continues to decline (Bullock et al., 2020; Hansen et al., 2020; Lee et al., 2014; Veldman et al., 2015). The decline in forest quality threatens the survival of many species and reduces forest service functions (Geldmann et al., 2013; Veldman et al., 2015; Ward et al., 2020). The concept of forest health has become increasingly important for sustainable forest use and development and is now a focal point of ecology and forestry research worldwide (Gauthier et al., 2015; Isabel et al., 2020; Millar & Stephenson, 2015; Trumbore et al., 2015; Watson et al., 2018). During the 1980s, a number of forest health‐related theories were investigated, assessed, and monitored in the United States and Germany (Trumbore et al., 2015). The forest health monitoring in the United States mainly involves three aspects: baseline survey, evaluation, and standing ecosystem coverage (Dash et al., 2017; White et al., 2013). European countries, on the contrary, emphasize the importance of environmental resources and biological information in assessing forest health (Lorenz & Mues, 2007; Requardt et al., 2009). Forest health assessment must also take into account various factors as well as the forest itself as a reference (Meng et al., 2019). In this regard, it is imperative to develop models that are capable of accurately assessing the health of forests in the future. Previous studies have used various methods to assess forest health, including principal component analysis (PCA), the health distance method (HDM), and comprehensive evaluation methods (Kayet et al., 2019; Tao et al., 2019; Xue et al., 2013; Zhu et al., 2013). However, all of these methods suffer from low classification accuracy, so some researchers have tried to solve this problem with machine learning algorithms (Ki, 2019). Meanwhile, several machine learning algorithms have been applied in various fields of research areas (Chen et al., 2021; Hansen et al., 2013; He et al., 2023; He, Zhao, Mao & Griffin‐Nolanb, 2022). Nevertheless, there is still considerable uncertainty about the effectiveness of forest health assessments. There are still gaps in the extent to which models provided by forest health indicators can accurately predict and assess the health of tropical rainforests.

It is inevitable that machine learning models for forest health assessments will produce uninterpretable results since machine learning itself is a “black box,” and accurate classification of forest health depends on credible and reliable models. Therefore, the innovation of the current study lies in its integrated approach to assessing forest health. It employs a broad suite of forest health indicators, capturing structural, compositional, and functional aspects of the forest ecosystem. Furthermore, the use of the decision tree algorithm, a machine learning technique, for modeling forest health represents a modern, data‐driven approach to ecological study. This method provides a robust and interpretable model for understanding the complex relationships between various indicators of forest health. Moreover, the use of the CRITIC method to determine the weights of different indicators based on their correlation offers an objective, quantitative way to prioritize different aspects of forest health. This approach ensures that the model's predictions are based on the most relevant and significant indicators. Together, these elements make this study a comprehensive, sophisticated, and innovative approach to assessing forest health in the WuZhi Shan area of Hainan Tropical Rainforest National Park.

## 2 MATERIALS AND METHODS

### 2.1 Study area

**FIGURE 1:**
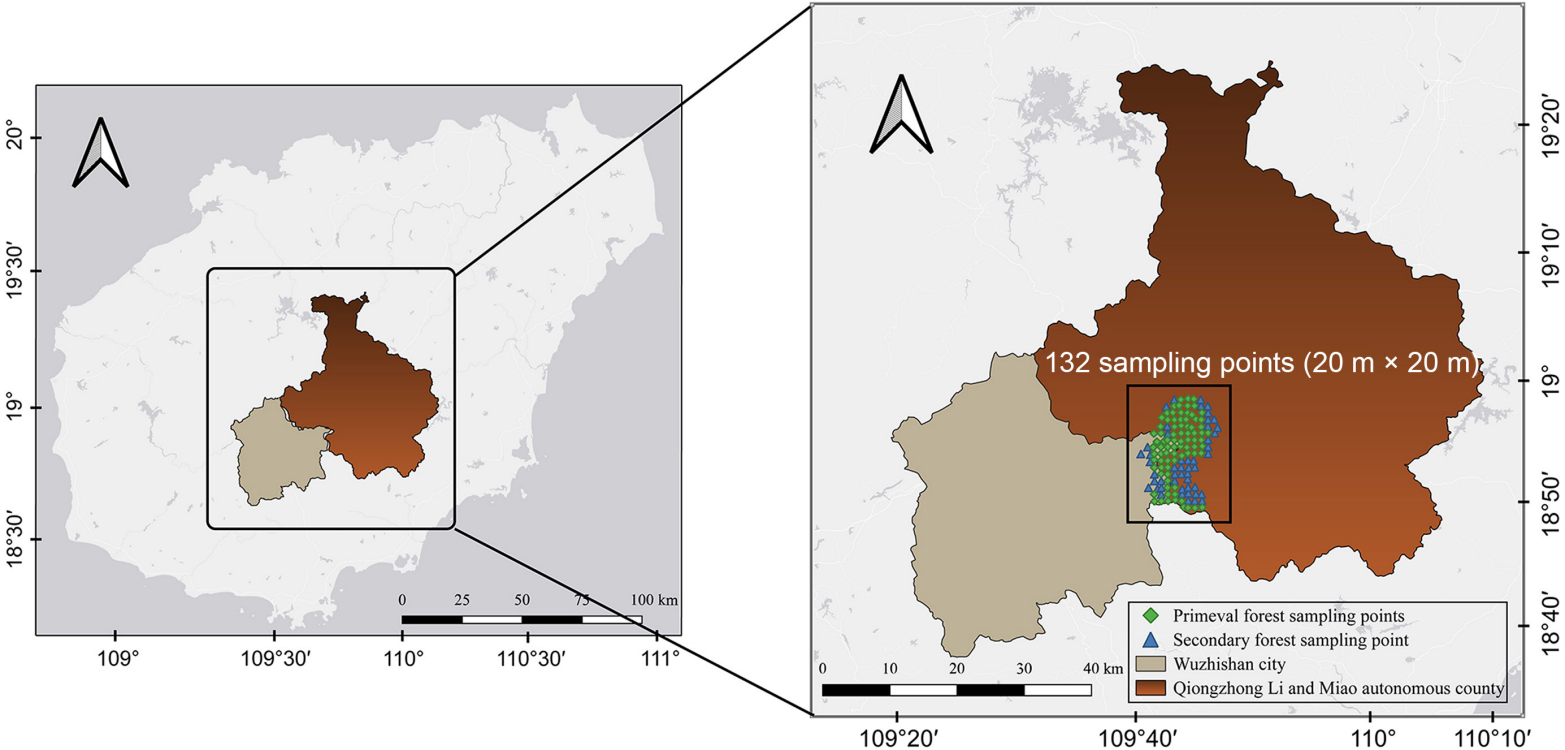
Study area and field sampling sites.

The study area is in the WuZhi Shan District of the Hainan Tropical Rainforest National Park (Hainan Province). For information on the specific characteristics of this study area, please see Table S1. The longitude of the study area ranges from 109.67 to 109.78 and the latitude from 18.82 to 18.97 (Figure 1). This study investigated the forest health of the WuZhi Shan from May 2020 to January 2021. A total of 132 forest samples were collected for this study, with each sample plot measuring 400 m^2^ (20 m by 20 m). Of these, 50 were secondary forest samples and 82 were primary forest samples, with a sample spacing of 1 km. The sample spacing of 1 km was chosen based on the size of the study area, the variability of the landscape, and the resolution of the data layer. This spacing allowed for a comprehensive sampling of the study area while keeping the total number of samples manageable.

### 2.2 Research dataset

In each sample, trees and shrubs with a diameter at breast height greater than or equal to 1.0 cm were painted and marked using a tape measure, and data such as species name, diameter at breast height, coordinates, and growth status were recorded, and a database was created. This study used GPS (UniStrong G128BD) to record the geographical coordinates of the sample plots and a compass to measure the direction of the slope of the sample plots. The slope of the sample plots was measured using a slope meter. Elevation data are derived from 12.5 m resolution Advanced Land Observing Satellite (ALOS) satellite data (Rosenqvist et al., 2007). Climate data were obtained at 1 km resolution from the WorldClim v. 2.1 database (Fick & Hijmans, 2017). Soil nutrient data were measured from soil samples. Five points of mixed soil samples were collected uniformly in the top 10 cm of each sample plot, shade‐dried, and ground to determine total phosphorus (TP), available phosphorus (AP), total nitrogen (TN), and available nitrogen (AN) content. We used ArcGIS 10.8 to resample all data layers to a common 30 m resolution (Scott & Janikas, 2010). In this study, we selected a comprehensive suite of 12 forest health indicators, representing structural, compositional, and functional aspects of the forest ecosystem (Table 1). These variables have Max (maximum value), Min (minimum value), Avg (average value), and SD (standard deviation), as shown in Table 2. These indicators collectively provide a multi‐faceted view of forest health, capturing the complexity and interdependence of forest attributes. The selection of these indicators was guided by their established relevance in forest ecology literature, as well as their potential to collectively offer a comprehensive and robust assessment of forest health (Augustin et al., 2009; Trumbore et al., 2015; Zarnoch et al., 2004). By incorporating a range of variables that span structural, compositional, and functional dimensions of the forest ecosystem, this study acknowledges the complex, multifactorial nature of forest health.

**TABLE 1:** The data of 12 variables were used in the study.

Notation	Description	Units
IL	Forest disturbance level	N/A
SDS	Slope direction and slope	°
DBH	Diameter at breast height	m
TH	Tree height	m
CWEW	Crown width (east and west)	m
CWNS	Crown width (north and south)	m
SNI	Soil impact index	N/A
FRI	Fire risk index	N/A
Menhinick	Species diversity index	N/A
Pielou	Uniformity index	N/A
Gleason	Forest richness index	N/A
Elevation	Elevation of the sampling site	m

**TABLE 2:** Results of statistical analysis of 12 variables.

Notation	Max	Min	Avg	SD
IL	1.00	0.38	0.13	0.15
SDS	1.00	0.38	0.20	0.24
DBH	5.16	1.92	0.52	0.67
TH	8.90	4.50	0.59	0.81
CWEW	3.68	2.18	0.28	0.35
CWNS	3.61	2.00	0.29	0.36
SNI	1.00	0.30	0.21	0.21
FRI	1.00	0.38	0.13	0.14
Menhinick	7.03	3.45	0.51	0.69
Pielou	3.65	2.09	0.28	0.35
Gleason	2.85	0.58	0.41	0.51
Elevation	1035.00	347.00	130.48	155.60

The Gleason diversity index was calculated as follows:

(1) $$D=\frac{S}{\ln N}$$

where S is the number of species recorded in the community and N the total area surveyed.

The Menhinick species richness index was calculated as follows:

(2) $$M=\frac{S}{\sqrt{n}}$$

where M is the Menhinick species richness index and n are the overall number of individuals of all species in the survey sample.

The Shannon‐Wiener index is calculated as follows:

(3) $$H=-\sum_{i=1}^{S}\;\left(P_{i}\log_{2}P_{i}\right)$$

where H is the Shannon‐Wiener index, $P_{i}$ is the proportion of individuals of the i species in the community or survey habitat among all the total.

The Pielou species evenness index was calculated as follows:

(4) $$\begin{array}{c} E=\frac{\left(-\sum_{i=1}^{S}\;P_{i}\log P_{i}\right)}{\log S} \end{array}$$

where E is the Pielou species evenness index. The Pielou species evenness index in equation (3) was calculated based on the Shannon‐Wiener index.

### 2.3 CRITIC analysis

This study used CRITIC method to calculate forest health (Diakoulaki et al., 1995; Konda & Tsitsiklis, 2003; Zahavy et al., 2020), which is a composite measure of the objective weight of each indicator based on the strength of contrast and inter‐indicator conflict of the selected indicators (Zahavy et al., 2020). The assessment was performed by considering the magnitude of variability and correlation between the indicators and by combining the objective properties of the data themself. The final evaluation results were used to calculate the forest health indicator (FH), which is used for training and validation of the decision tree model (Table S2).

The conflict between indicators was expressed by the correlation coefficient, and when the correlation between two indicators was high, the degree of conflict was smaller and the weight was lower. In the weighting operation, the contrast intensity was multiplied by the conflict property and then normalized to obtain the final weight.

CRITIC uses dimensionless processing to eliminate the impact on the results due to differences in magnitudes, as follows.

(5) $$\begin{array}{c} x_{\mathrm{ij}}^{\prime }=\frac{x_{j}-x_{\min}}{x_{\max}-x_{\min}} \end{array}$$

The variability of CRITIC's indicators is expressed in the form of standard deviation by the following mathematical procedure.

(6) $$\begin{array}{c} \left\{\begin{array}{c} {\bar{x}}_{j}=\frac{1}{n}\sum_{i=1}^{n}\;x_{\mathrm{ij}}\\ S_{j}=\sqrt{\frac{\sum_{i=1}^{n}\;{\left(x_{\mathrm{ij}}-{\bar{x}}_{j}\right)}^{2}}{n-1}} \end{array}\right. \end{array}$$

where $S_{j}$ denotes the standard deviation of the j indicator.

The conflicting nature of the indicators can be expressed in terms of correlation coefficients using the following mathematical procedure.

(7) $$\begin{array}{c} R_{j}=\sum_{i=1}^{p}\;\left(1-r_{\mathrm{ij}}\right) \end{array}$$

where $r_{\mathrm{ij}}$ represents the correlation coefficient between evaluation indicators i and j.

The correlation coefficient was used to express the correlation between the selected indicators. The stronger the correlation between indicators, the lower the conflict between the indicators, then there will be more repetitions in the evaluation, and the intensity of the assessment would be weakened, for which the amount of information of the assigned weights would be reduced, and the specific mathematical operations as follows.

(8) $$\begin{array}{c} C_{j}=S_{j}\sum_{i=1}^{p}\;\left(1-r_{\mathrm{ij}}\right)=S_{j}\times R_{j} \end{array}$$

where the greater the $C_{j}$, the greater the role of the $C_{j}$ evaluation indicator in the evaluation index system, the more weight should be assigned to it. So, the objective weight $W_{j}$ of the j indicator is expressed as:

(9) $$\begin{array}{c} W_{j}=\frac{C_{j}}{\sum_{j=1}^{p}\;C_{j}} \end{array}$$

### 2.4 Decision tree model

**FIGURE 2:**
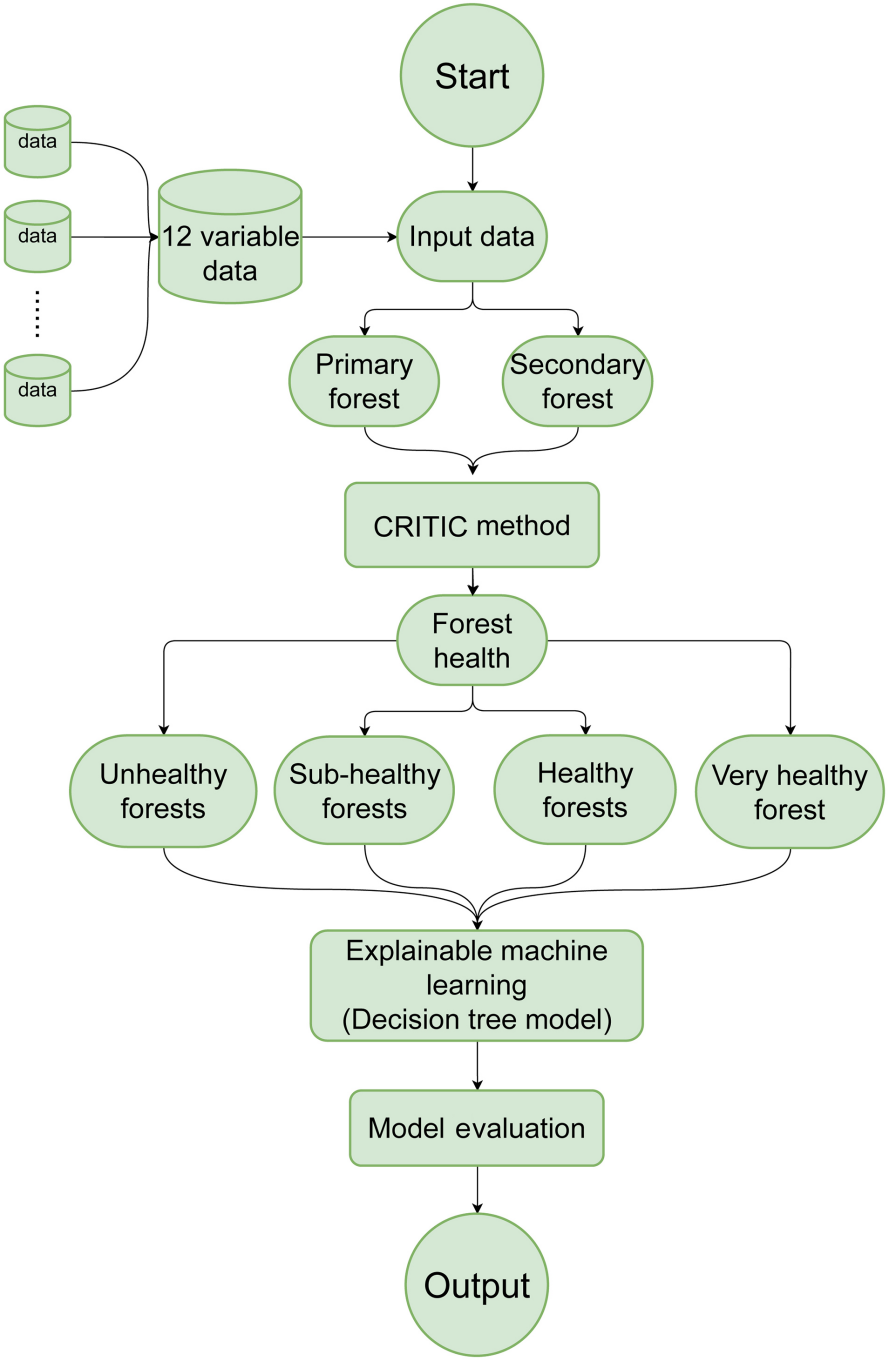
Explainable forest health assessment workflow.

Decision trees are very powerful and popular machine learning algorithms that are widely used as a predictive model for practical applications in many fields (Chen et al., 2018, 2020; Kotsiantis, 2013; Linero, 2018; Lundberg et al., 2020; Yariyan et al., 2020). Since the size of a decision tree is independent of the size of the dataset, it is possible to construct decision trees from a large number of datasets with multiple attributes (Kingsford & Salzberg, 2008). Decision tree construction algorithms can first create the tree and then prune it to achieve more efficient classification. Entropy is used to describe the uncertainty of information (Kingsford & Salzberg, 2008). This implementation uses an optimized version of the CART (Classification and Regression Trees) algorithm. The model was used with default parameters, including a Gini criterion to measure the quality of a split and a best‐first strategy for node splitting until the leaves are pure or until they contain less than two samples. This implementation provided an effective means of modeling the complex relationships between various indicators of forest health. The workflow flow chart of the study is shown in Figure 2.

The purpose of decision tree classification is to iteratively divide a given data set into subsets where all elements in each final subset belong to the same class. The entropy is calculated as shown in equation (10).

(10) $$\begin{array}{c} H\left(D\right)=-\sum_{k=1}^{K}\;\frac{\left|C_{k}\right|}{\left|D\right|}\log_{2}\frac{\left|C_{k}\right|}{\left|D\right|} \end{array}$$

where D denotes the sample size and K denotes how many classes are in the sample.

The entropy formula for D for a given condition of feature A is expressed as:

(11) $$\begin{array}{c} H\left(D|A\right)=\sum_{i=1}^{n}\;\frac{\left|D_{i}\right|}{\left|D\right|}H\left(D_{i}\right) \end{array}$$

where feature A has n different values (a1, a2… an), and D is divided into n subsets (D1, D2… Di) according to the values of feature A. The number of samples of $D_{i}$.

The formula for the information gain of feature A on training D is expressed as follows:

(12) $$\begin{array}{c} g\left(D,A\right)=H\left(D\right)-H\left(D|A\right) \end{array}$$

The ratio of the information gain of feature A to training D is expressed by the following equation.

(13) $$\begin{array}{c} g_{R}\left(\mathrm{DA}\right)=\frac{g\left(\mathrm{DA}\right)}{H\left(D\right)} \end{array}$$

When the training data contain a lot of noise, the accuracy would be too low. Each classifier is assigned a vote and will be assigned to the class with the highest number of votes. Classification and regression tree (CART) is the process of generating a binary tree for decision making (Quinlan, 1986). CART handles the missing data and includes pruning strategies. The algorithm selects the best partition point according to gini value, which is calculated as follows.

(14) $$\begin{array}{c} \text{gini}\left(D\right)=1-\sum_{k=1}^{K}\;{\left(\frac{\left|C_{k}\right|}{\left|D\right|}\right)}^{2} \end{array}$$

The formula for the gini value of feature A for training set D is expressed as follows:

(15) $$\begin{array}{c} \text{gini}\left(D,A\right)=\frac{\left|D_{1}\right|}{\left|D\right|}\text{Gini}\left(D_{1}\right)+\frac{\left|D_{2}\right|}{\left|D\right|}\text{Gini}\left(D_{2}\right) \end{array}$$

To assess the accuracy of our decision tree model, we computed several metrics: precision, recall, F1 score, and the area under the Receiver Operating Characteristic curve (AUC) (Raschka, 2018). Precision provides a measure of result relevancy, while recall provides a measure of how many truly relevant results are returned. The f1 score provides a balance between precision and recall. AUC, in contrast, measures the model's ability to distinguish between different classes across various threshold settings.

(16) $$\begin{array}{c} \text{Precision}=\frac{\;\text{True positives}\;}{\;\text{True positives}+\text{False positives}\;} \end{array}$$

(17) $$\begin{array}{c} \text{Recall}=\frac{\;\text{True positives}\;}{\;\text{True positives}+\text{False negatives}\;} \end{array}$$

(18) $$\begin{array}{c} \text{f1}=2\times \frac{\;\text{Precision}\times \text{Recall}\;}{\;\text{Precision}+\text{Recall}\;} \end{array}$$

(19) $$\begin{array}{c} \mathrm{AUC}=\frac{\sum_{i=1}^{n_{1}+n_{2}}\;\left(y_{i}\cdot {\mathrm{rank}}_{i}\right)-n_{1}\times \frac{\left(n_{1}+1\right)}{2}}{n_{1}\cdot n_{2}} \end{array}$$

where ${\mathrm{rank}}_{i}$ is the rank of the i prediction in the sorted list. For a set of predictions sorted by their predicted probabilities in descending order, where $y_{i}$ is the true label (1 for positive, 0 for negative) and $n_{1}$ is the number of positive instances, $n_{2}$ is the number of negative instances.

All experiments in this study and the construction of the decision tree model were run on a computer with a 64 for Window 10 operating system, an Intel(R) Core (TM) i7‐10,700 2.9GHz processor and 16GB. The models were constructed by Python 3.8 version programming language with scikit‐learn machine learning library composition (Pedregosa et al., 2011; Van Rossum & Drake, 2009), all hyperparameters were used with default settings, and randomly selected 80% of the data was selected for training and 20% of the data was used for validation (Joseph, 2022).

## 3 RESULTS

### 3.1 Correlation analysis of indicators

**FIGURE 3:**
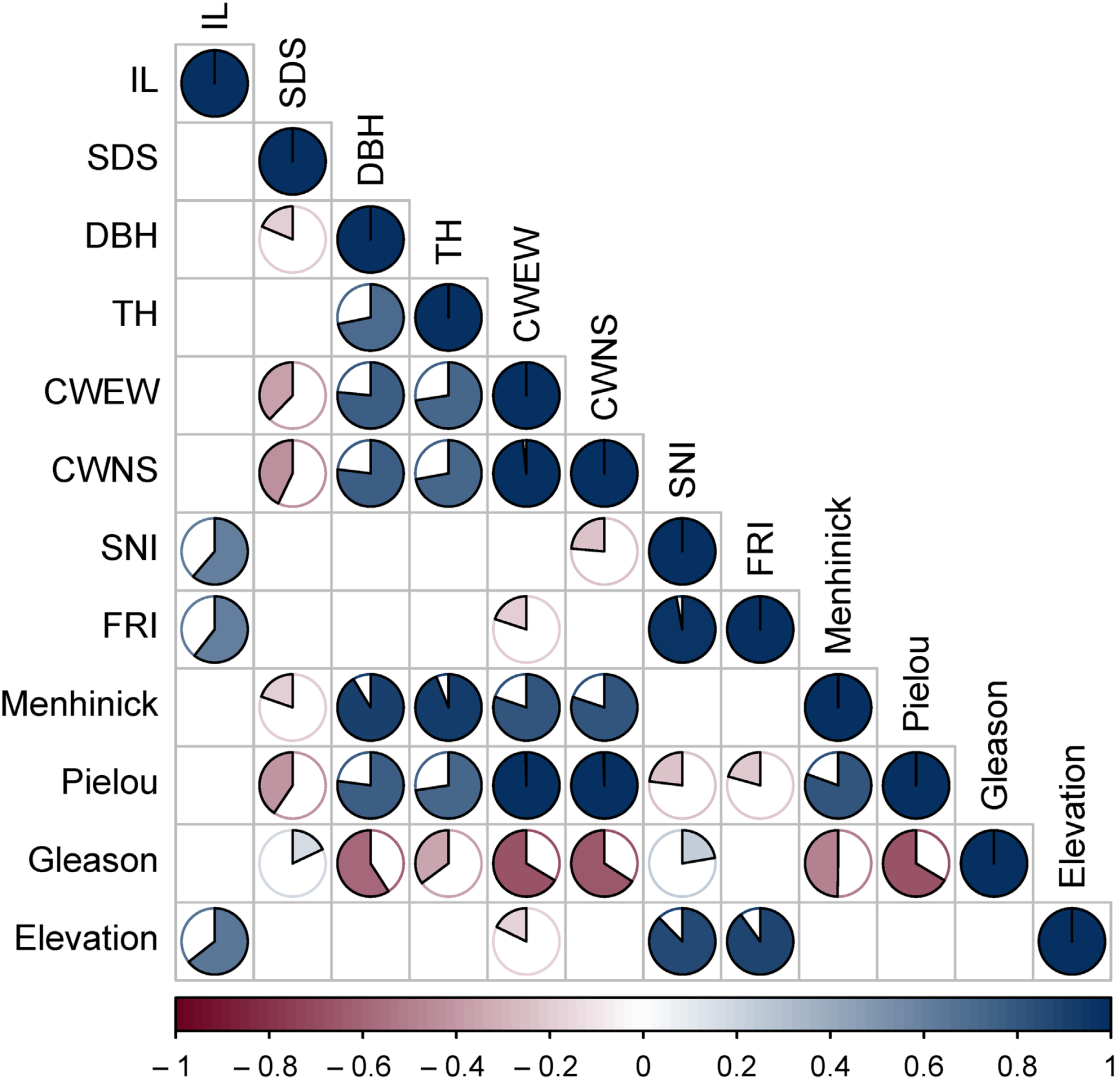
Correlation analysis between indicators. Refer to Table 1 for an explanation of the acronyms.

Figure 3 elucidates the intricate interrelationships among the forest health indicators, providing a comprehensive view of the underlying correlations. The colors in the figure indicated the positive and negative correlations, the area of the circle showed the strength of the correlation coefficient, and the area not shown meant no statistical significance (*p* > .05). Among them, the correlations between CWNS and CWEW and the Pielou index were high (0.996 and 0.995, respectively), and Pielou increased with the increase in canopy width; the correlation between SNI and FRI was 0.971; and the correlation between Menhinick and TH was 0.940.

### 3.2 CRITIC weighting analysis

**FIGURE 4:**
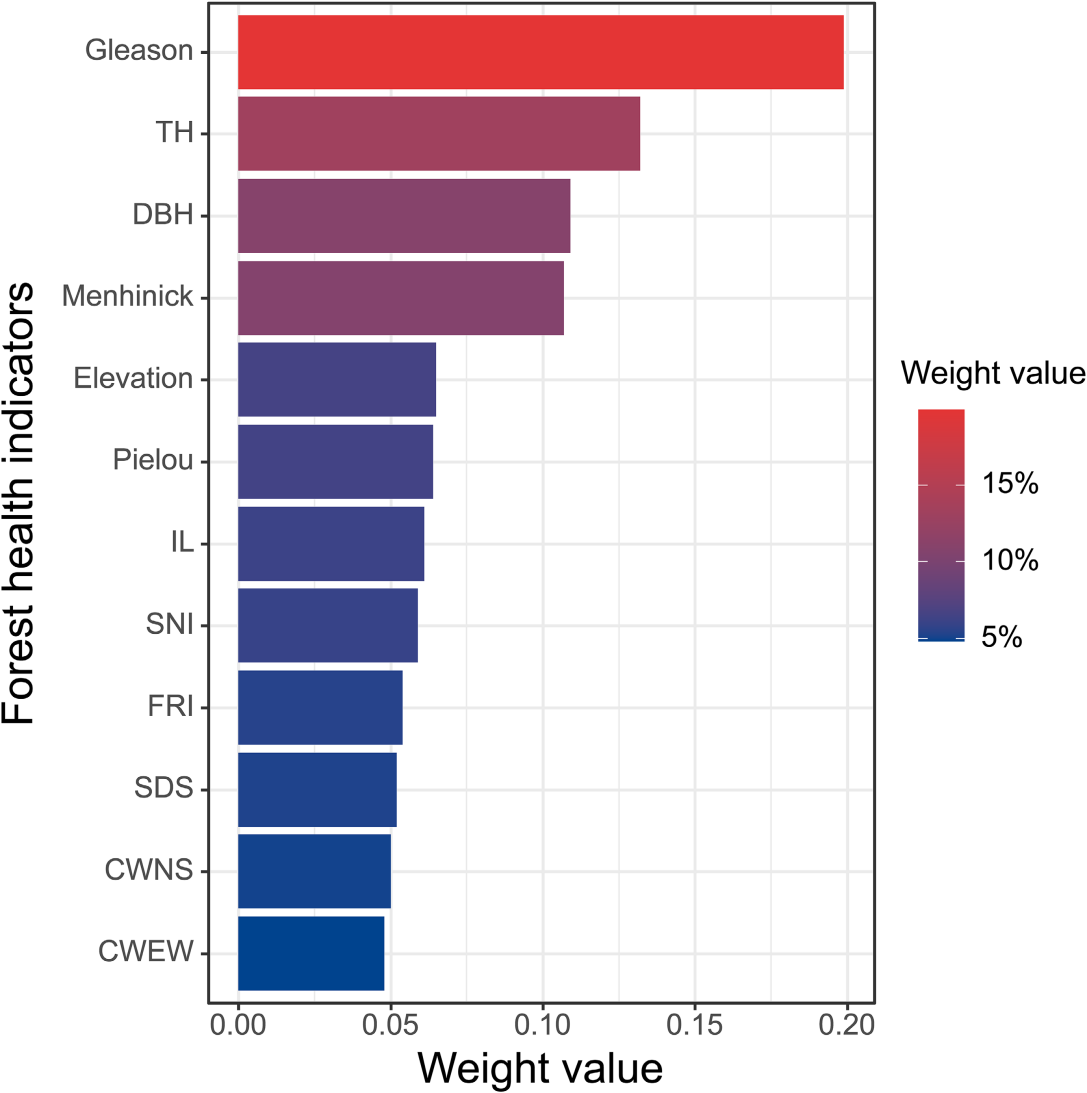
CRITIC weight assignment results. Refer to Table 1 for an explanation of the acronyms.

The outcomes of the CRITIC weighting analysis are encapsulated in Figure 4, quantitatively delineating the relative significance of each indicator. The Gleason diversity index was found to have the highest weight of 19.9%, which also indicates that species diversity is extremely important for forest health. The next highest weight was given to TH with 13.2%, followed by DBH with 10.9%, while other indicators also contributed more than 5%. It is clear from the results that each indicator has a certain reference value and is essential for the forest health assessment.

### 3.3 Decision tree model classification

**FIGURE 5:**
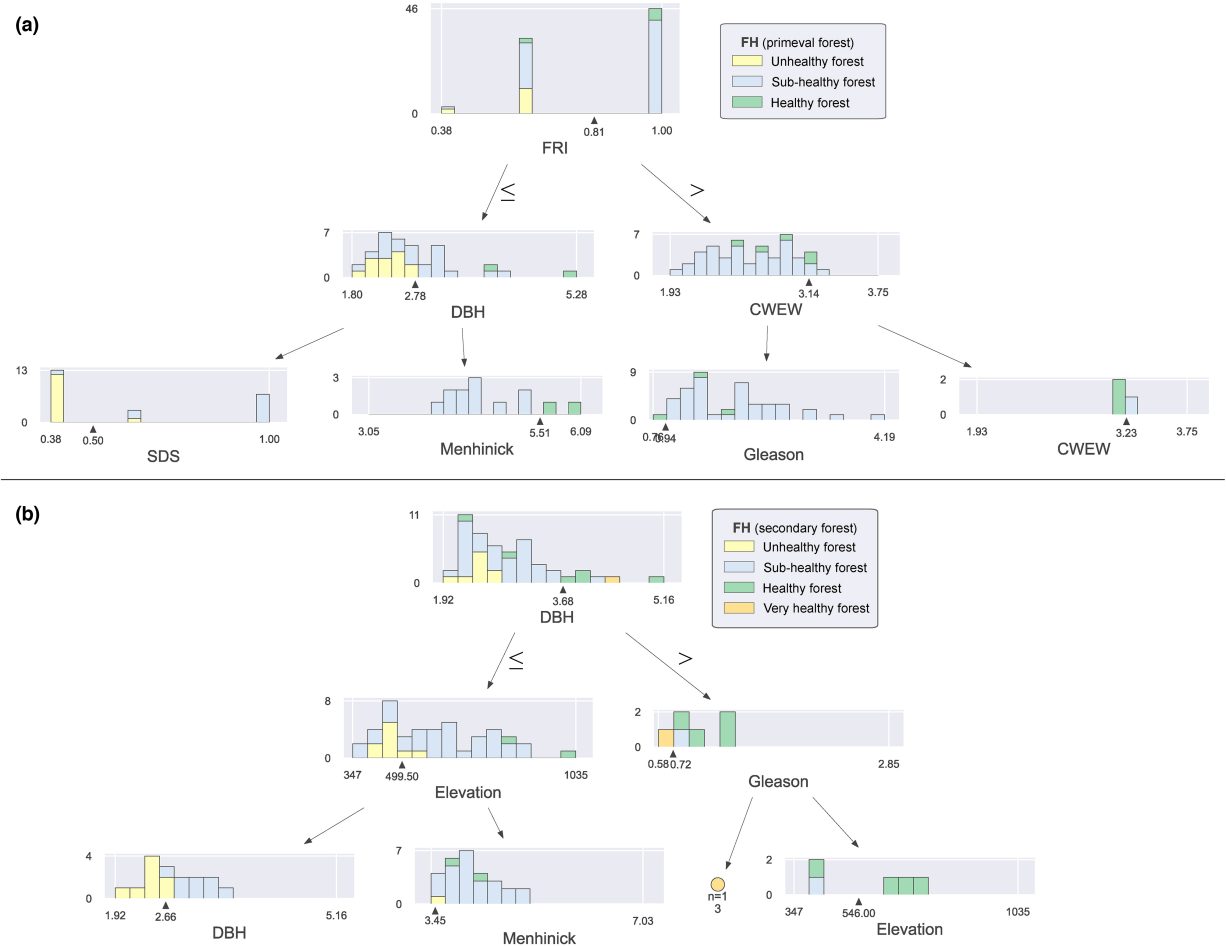
Decision tree model for forest health status. Panel (a) shows the forest health classification of primary forest and panel (b) shows the forest health classification of secondary forest. Refer to Table 1 for an explanation of the acronyms.

Figure 5 offers a graphical representation of the decision tree model, facilitating an intuitive comprehension of the model's decision‐making process. In this study, the maximum depth of the decision tree model was set to 3, to better display the model results. The complete model can be found in the Supplementary Appendix (Figure S1). The results showed that the decision tree model can reasonably classify forest health conditions based on the input variables, which illustrates that the machine learning model can be used in forest health evaluation and provide reasonable explanations.

### 3.4 Explainable models

**FIGURE 6:**
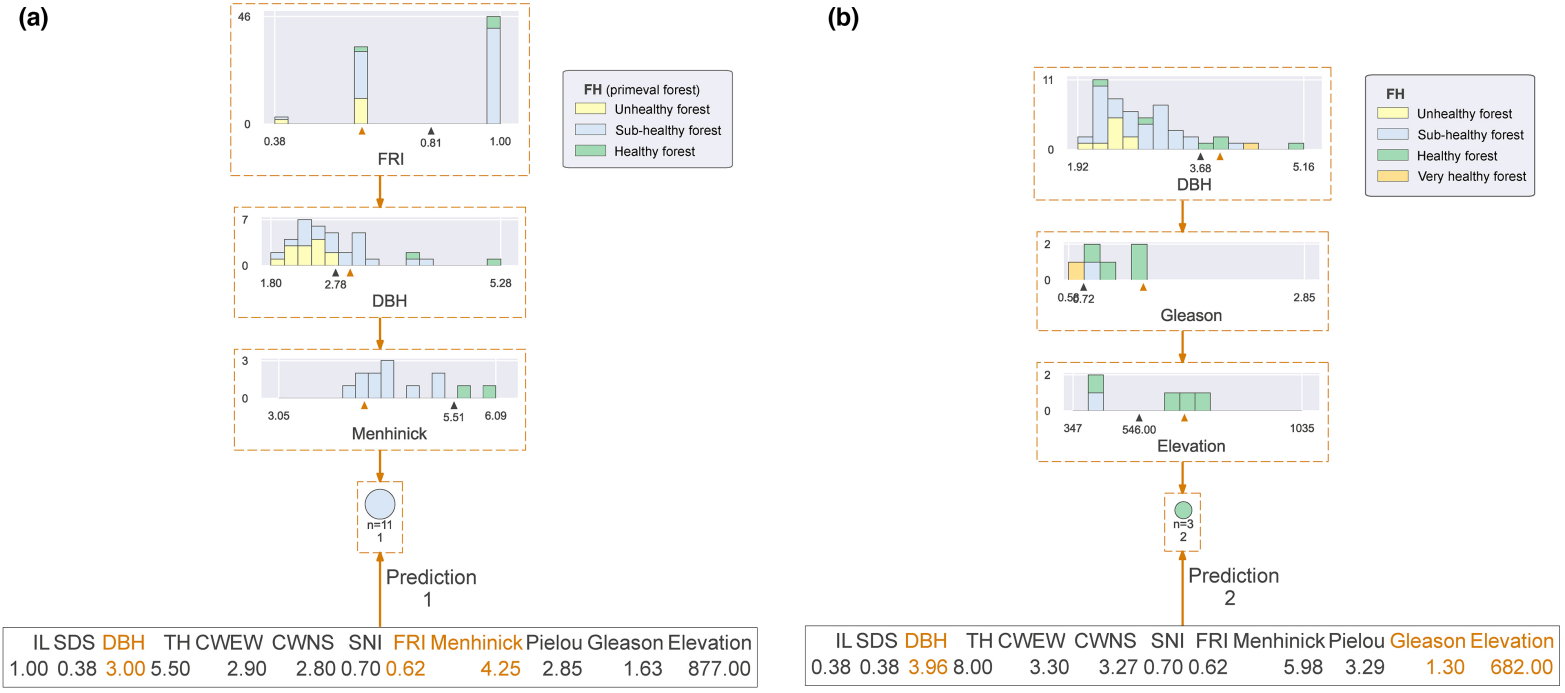
Explainable decision tree model. Panel (a) shows the forest health classification of primary forest and panel (b) shows the forest health classification of secondary forest. Refer to Table 1 for an explanation of the acronyms.

To better represent the generalization of the model, we elaborated the model using interpretable machine learning methods. The complete structure of the decision tree model is explained in the Supplementary Appendix (Figure S2). The results showed that the classification of primary forest health was mainly determined by the variables FRI, DBH, and Menhinick (Figure 6). In contrast, the classification of the health status of secondary forests was determined by DBH, Gleason, and Elevation. It can be seen that the FRI is not the most critical variable for secondary forests, although it strongly influences the health status of primary forests. However, the DBH variable is an important factor that is essential for the health status of both primary and secondary forests.

### 3.5 Model evaluation

**FIGURE 7:**
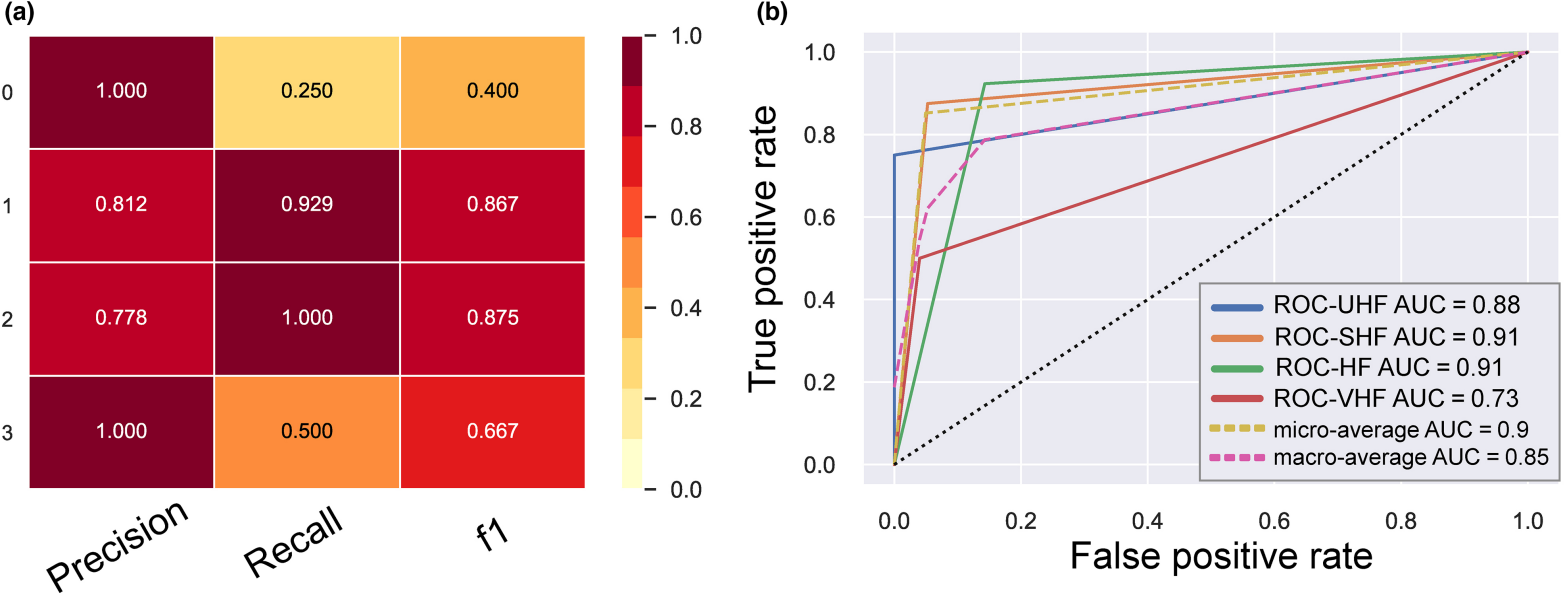
Integrated assessment of forest health models. Where UHF (0) indicates an unhealthy forest, SHF (1) indicates a sub‐healthy forest, HF (2) indicates a healthy forest, and VHF (3) indicates a very healthy forest.

In this study, a comprehensive evaluation of the model was carried out to better reflect the rationality of the decision tree model's classification. The evaluative metrics of the model's performance, including precision, recall, and F1 score, are succinctly compiled in Figure 7, offering a robust assessment of the model's predictive efficacy. The decision tree model performed well in predicting the forest health classes. The average precision of the model was 0.8975, the recall was 0.66975, and the f1 score was 0.70225. These values indicate a strong performance of the model in both identifying true positives and limiting false positives in Figure 7a. However, it can be found that the “unhealthy forest” and “very healthy forest” classifications do not perform well on the recall and f1 evaluation metrics, while the precision values perform better. The receiver operating characteristic curve (ROC) results of the model are shown in Figure 7b, and the ROC results clearly show the sample identification ability of the forest health model. The AUC results of the model showed an overall high index (average AUC = 0.9). This indicates that the decision tree model can correctly classify forest health.

## 4 DISCUSSION

Keeping forests healthy is of utmost importance, and great care should be taken to preserve their value. A key aspect of environmental policy and resource management is the assessment and monitoring of forest health at all stages of the forest lifecycle. In this study, the decision tree algorithm was used to develop the modeling, which has the advantage of high accuracy and interpretability (AUC = 0.9). Among all the indicators, the Gleason diversity index, tree height, and diameter at breast height had the highest CRITIC weight values. Tree height is a factor that responds to the tall‐ and short‐growth status of trees and also has a major impact on forest quality. Due to competition for sunlight, trees with greater heights flourish, while trees with smaller heights are suppressed and ultimately die off. In addition, it affects shade in streams and the understory vegetation over time. The results showed that Gleason diversity index was found to have the highest weight, indicating that species diversity is extremely important for forest health. Because there are more than 70,000 species of plants and animals on Earth and the forest provides a wide range of ecosystem goods and services to humanity (Pan et al., 2013). Species traits play an important role in determining ecosystem functions and processes, and forest biodiversity is the foundation for many ecosystem services (Mori et al., 2017).

The diameter at breast height is highly correlated with many other indicators and is an important indicator for forest inventory, as well as an important basis for calculating forest stock and measuring the forest trees' growth status. The elevational distribution of species richness in nature is uneven and varies according to various factors such as latitude, altitude, precipitation, temperature, and study area landscape. Elevation plays an important role in predicting tree species distribution in mountain ecosystems of central Europe. There is evidence that geomorphometric variability influences species occurrence and biomass, resulting in higher species diversity (Dyderski & Pawlik, 2020). However, species diversity is spatially distributed differently at different scales due to different controlling factors (Stein et al., 2014). The interactions between these factors, including natural and biological factors, and their ecological processes are more complex and diverse, and species richness also has a greater impact on forest health. Many tropical forest studies have highlighted soil moisture regimes' importance in determining plant diversity distributions (Bonetti et al., 2017; Engelbrecht et al., 2007; Givnish, 1999).

It is important to assess forest health by considering the slope gradient and slope orientation, which are both important indicators of soil, water content, and forest surface water runoff. Additionally, slope orientation can directly affect light levels in the forest. As well as serving as a reference target for establishing the growth rate of trees, the canopy also serves as an important site for photosynthesis and tree respiration. The width of the canopy in the north–south direction and in the east–west direction directly affects tree vigor and productivity, which in turn affects forest health. The degree of disturbance and the recovery time after disturbance directly affect the quality of the forest. The WuZhi Shan district's forests include both natural and secondary forests formed after logging, with varying degrees of disturbance and recovery. Forest ecosystems are constantly subjected to a variety of disturbances since both natural processes and human activities are involved, particularly inappropriate exploitation, which leads to the degradation of forest ecosystems around the world. The loss and accumulation of soil nutrients in tropical forests depend on the relative strengths of bioaccumulation and decomposition, the intensity of leaching, hydrothermal conditions, and microbial activities. Second, human activities can also have a large impact on the forest floor ecosystem to some extent, leading to the destruction of forest health.

In this study, the decision tree model served as a robust machine learning classification tool for assessing forest health, providing interpretability, versatility in data handling, and an insightful understanding of feature importance. While potential drawbacks such as overfitting and instability were considered, these were mitigated through diligent model tuning and cross‐validation. Even though minor alterations in data could impact the tree structure, the transparency and ability of the model to handle complex, high‐dimensional datasets effectively outweighed this limitation. As corroborated by a previous study (Kotsiantis, 2013), machine learning techniques like decision trees offer reliable predictions of forest health indicators, enabling us to simultaneously consider multiple health indicators and understand their relative importance. Despite the noted limitations, the careful implementation of our decision tree model ensured robust and reliable outcomes. In alignment with the core thesis of this research—that a diverse suite of forest health indicators can be robustly interrogated through machine learning methodologies to ascertain the health status of the WuZhi Shan area—we elected to utilize the decision tree model. This selection was underpinned by the model's inherent capacity to harmonize interpretability with versatility while maintaining a satisfactory degree of predictive accuracy. The transparency proffered by decision trees rendered a lucid representation of how our delineated indicators were leveraged to generate predictions, a facet indispensable to our ecological investigation. Unlike alternative models, such as Support Vector Machines or Random Forests, decision trees have the capacity to handle heterogeneous data types and do not predicate on assumptions about predictor distributions, thus aligning seamlessly with our diversified dataset. While ensemble methods like Random Forests might potentially offer enhanced predictive accuracy, they introduce complexity to interpretability, thereby complicating the comprehension of relationships between predictors—a fundamental objective of our study. Similarly, while Multivariate Adaptive Regression Splines possess the ability to model nonlinear relationships, their increased complexity and diminished interpretability render them less suitable for our investigative purposes. Therefore, our choice of the decision tree model was instrumental in furthering our core thesis by offering an optimal amalgamation of interpretability, flexibility, and predictive capability.

Forest health assessments continue to have significant limitations. Information on forest health is often inadequate (Trumbore et al., 2015). Because the information required to measure forest health is particularly extensive, it is difficult to accurately calculate spatial diversity and forest mortality, although more information is available through remote sensing (Guimaraes et al., 2020; Iglhaut et al., 2019; White et al., 2013). The data we used was based on a sample of 132 forests, so we can build decision tree models efficiently and accurately, but when assessing forest health at larger scales, decision tree models can have poor fitting and other problems. It is recommended to use a heuristic algorithm and optimize the decision tree algorithm, using a decision tree‐based combination algorithm to solve the problems of overfitting and increasing error rates (He, Jia et al., 2022). The forest health calculated in this study using 12 indicators is still not the most accurate representation of actual forest health; further studies are needed regarding the implementation of more indicators such as climate, forest productivity, and ecological indicators. In our opinion, forest health classification is particularly important for regional forest protection because it may identify and diagnose the health of the forest from the ground up for targeted conservation and protection. Currently, available techniques for measuring forest health have major gaps and cannot provide all the information needed to systematically assess changes in forest health. Future research could address these limitations by incorporating more localized and detailed data, exploring more complex modeling approaches, and comparing results across different forest types and regions.

## 5 CONCLUSION

In the current study, a machine learning model was used to construct a forest health assessment of primary and secondary forests in the study area. Twelve indicators were used to assess forest health by combining the forest kilometer grid survey. The objective weights of CRITIC were used to calculate the health status of primary and secondary forests in the study area, and it was demonstrated that each indicator has more or less influence on forest health. Moreover, this study proposes a decision tree model to solve the unexplained problem in forest health assessment by visualizing the information of data indicators and opening the “black box” of machine learning. Study results showed that decision tree models can accurately classify forest health conditions. The most influential indicator of the model to classify the health status of primary and secondary forests is the diameter at breast height, but the most important indicator for forest health assessment is the Gleason index. A forest health assessment model based on decision trees would be useful for sustainable forest conservation and targeted forest health restoration.

## AUTHOR CONTRIBUTIONS

**Jialing Li:** Conceptualization (equal); project administration (equal); resources (equal); writing – review and editing (equal). **Bohao He:** Conceptualization (lead); data curation (equal); formal analysis (lead); methodology (lead); software (lead); validation (lead); visualization (lead); writing – original draft (lead); writing – review and editing (equal). **Shahid Ahmad:** Supervision (equal); validation (equal); writing – review and editing (equal). **Wei Mao:** Conceptualization (equal); funding acquisition (equal); project administration (equal); resources (equal); supervision (equal); validation (equal); writing – review and editing (equal).

## FUNDING INFORMATION

This research was funded by the Major Science and Technology Project of Hainan Province (ZDKJ202008), the Start‐up funding from Hainan University (kyqd20035).

## CONFLICT OF INTEREST STATEMENT

The authors declare that they have no known competing financial interests or personal relationships that could have appeared to influence the work reported in this paper.

## Supporting information


Appendix S1:

## Data Availability

The Data that support the findings of this study are openly available in Dryad at: https://doi.org/10.5061/dryad.qrfj6q5jm.
