# Supplementary material for: Leveraging explainable machine learning models to assess forest health: A case study in Hainan, China
Source: Ecol Evol. 2023 Sep 25;13(9):e10558. doi: 10.1002/ece3.10558 (PMC10518842; doi:10.1002/ece3.10558)
Supplement: Supplementary file 1 — Appendix S1: [file ECE3-13-e10558-s001.docx]

**Supplementary information**

**Leveraging explainable machine learning models to assess forest health: A case study in Hainan, China**

**
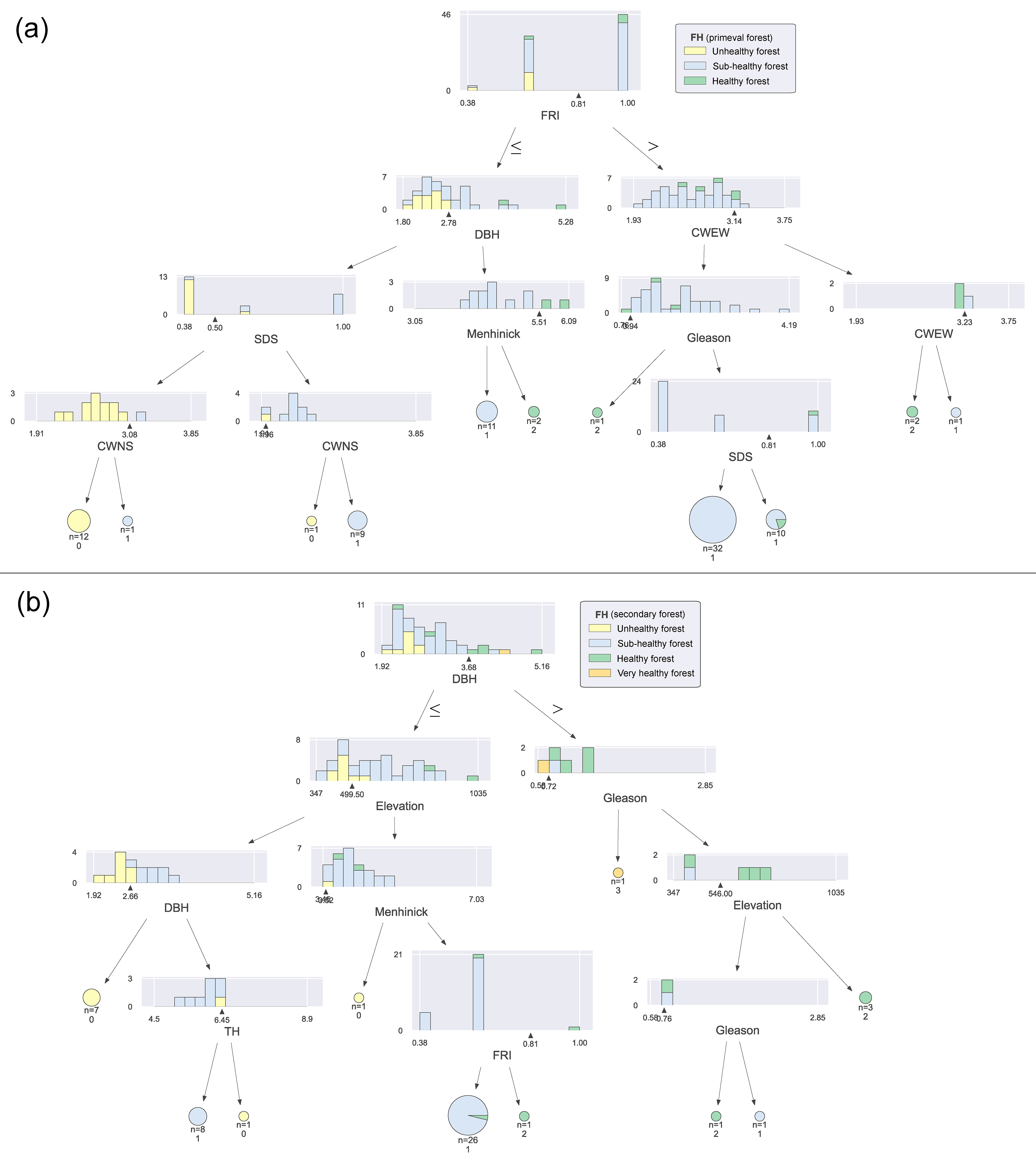
**

Figure. A1. Decision tree model complete branch structure. Figure A1 (a) showed the forest health classification of primary forest and Figure A1 (b) showed the forest health classification of secondary forest.


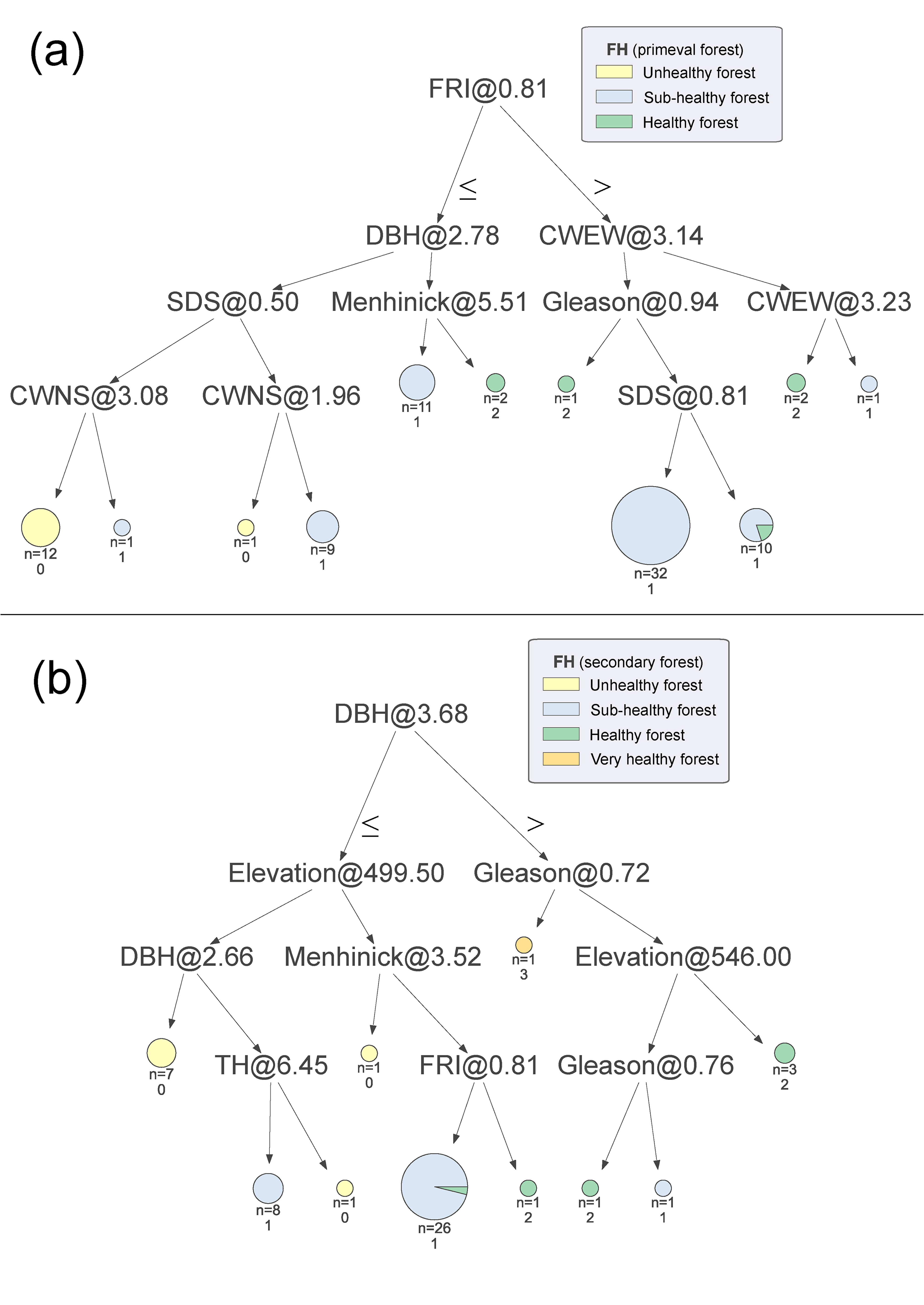


Figure. A2. The complete structure of the decision tree model is interpretable. Figure A2 (a) showed the forest health classification of primary forest and Figure A2 (b) showed the forest health classification of secondary forest.

Table A1. The regional characterization profile of the study.

| Parameter | Details |
| --- | --- |
| Study Area | WuZhi Shan area of Hainan Tropical Rainforest National Park |
| Geographical Coordinates | Between N18°53′–19°10′ latitude and E109°32′–109°42′ longitude |
| Elevation Range | Ranging from 934.1 m to 1413.6 m |
| Climate | Tropical monsoon climate |
| Average Annual Temperature | 23.2 °C |
| Extreme Maximum Temperature | 38.1 °C |
| Extreme Minimum Temperature | -1.8 °C |
| Average Annual Precipitation | 1,924.9 mm |
| Maximum Monthly Precipitation | 392.4 mm |
| Minimum Monthly Precipitation | 3.1 mm |
| Average Annual Relative Humidity | 85% |
| Sample Plots | 132 sample plots |
| Plot Size | Each plot 20 m x 20 m |
| Data Collection Time | From September to December 2022 |
| Training | All team members trained to ensure consistency |

Table A2. The forest health of 132 sample plots in the study area. The data became the target variable used to construct a decision tree model.

| Forest type | Forest Health | Number |
| --- | --- | --- |
| Primeval forest | Unhealthy forests | 0 |
|  | Sub-healthy forests | 13 |
|  | Healthy forests | 62 |
|  | Very healthy forest | 7 |
| Secondary forest | Unhealthy forests | 9 |
|  | Sub-healthy forests | 34 |
|  | Healthy forests | 6 |
|  | Very healthy forest | 1 |
